# Step by step: reconstruction of terrestrial animal movement paths by dead-reckoning

**DOI:** 10.1186/s40462-015-0055-4

**Published:** 2015-09-15

**Authors:** O. R. Bidder, J. S. Walker, M. W. Jones, M. D. Holton, P. Urge, D. M. Scantlebury, N. J. Marks, E. A. Magowan, I. E. Maguire, R. P. Wilson

**Affiliations:** Institut für Terrestrische und Aquatische Wildtierforschung, Stiftung Tierärztliche Hochschule, Hannover, Werfstr. 6, 25761 Büsum, Germany; Department of Computer Science, College of Science, Swansea University, Singleton Park, Swansea, SA2 8PP, Wales UK; College of Engineering, Swansea University, Singleton Park, Swansea, SA2 8PP Wales UK; Faculté des Sciences de la Vie, Master d’Ecophysiologie et Ethologie, Université de Strasbourg, 28 rue Goethe, 67083 Strasbourg Cedex, France; School of Biological Sciences, Institute for Global Food Security, Queen’s University Belfast, Belfast, BT9 7BL Northern Ireland UK; Swansea Lab for Animal Movement, Biosciences, College of Science, Swansea University, Singleton Park, Swansea, SA2 8PP Wales UK

**Keywords:** Step length, dead reckoning, animal movement, GPS, terrestrial

## Abstract

**Background:**

Research on wild animal ecology is increasingly employing GPS telemetry in order to determine animal movement. However, GPS systems record position intermittently, providing no information on latent position or track tortuosity. High frequency GPS have high power requirements, which necessitates large batteries (often effectively precluding their use on small animals) or reduced deployment duration. Dead-reckoning is an alternative approach which has the potential to ‘fill in the gaps’ between less resolute forms of telemetry without incurring the power costs. However, although this method has been used in aquatic environments, no explicit demonstration of terrestrial dead-reckoning has been presented.

**Results:**

We perform a simple validation experiment to assess the rate of error accumulation in terrestrial dead-reckoning. In addition, examples of successful implementation of dead-reckoning are given using data from the domestic dog *Canus lupus,* horse *Equus ferus*, cow *Bos taurus* and wild badger *Meles meles*.

**Conclusions:**

This study documents how terrestrial dead-reckoning can be undertaken, describing derivation of heading from tri-axial accelerometer and tri-axial magnetometer data, correction for hard and soft iron distortions on the magnetometer output, and presenting a novel correction procedure to marry dead-reckoned paths to ground-truthed positions. This study is the first explicit demonstration of terrestrial dead-reckoning, which provides a workable method of deriving the paths of animals on a step-by-step scale. The wider implications of this method for the understanding of animal movement ecology are discussed.

**Electronic supplementary material:**

The online version of this article (doi:10.1186/s40462-015-0055-4) contains supplementary material, which is available to authorized users.

## Background

Animal movement interests animal biologists because, *inter alia*, it determines the success of individuals in obtaining resources, avoiding predation, maximising fitness and managing energetic profitability [1–3]. The success of individuals modulates populations and drives evolution and the diversity of life [4]. There are also numerous practical benefits to understanding animal movement, such as predicting the impact of land use changes, control of invasive and pest species, conservation of endangered species and foreseeing the spread of zoonotic diseases [5–8].

Obtaining the required information on animal movements is far from trivial, however, as many species operate in environments that preclude them from being observed (e.g. [9, 10]). Many biotelemetry methods deal with this [11] because they obviate the need for visual contact between researcher and study animal. The two methods most frequently applied in terrestrial environments for obtaining animal location data are VHF and GPS telemetry [12, 13]. Both however, have their limitations [14]; VHF is an established method, but requires significant field effort to implement [15] while GPS telemetry is considered to be ‘accurate’ [16] but prone to bias according to the environment [17, 18], particularly with regard to vegetation [19] and landscape topography [20]. In addition, the high current drain of GPS systems necessitate large batteries when recording at high sampling rates, which limits use on smaller species [21–23], or restricts researchers to deployments of shorter duration [24]. Analysis of data obtained by both methods assumes straight line travel between temporally infrequent positions [25] even though much animal movement is known to be highly tortuous [26]. Clearly, there is a need for fine-scale animal movement data in both space and time so that animal movement models can better reflect the true nature of animal movement (c.f. [27]).

In fact, the only biotelemetric method purported to produce fine scale (i.e. >1 Hz) terrestrial animal movement data is dead-reckoning [28–30] which may resolve movement so finely that it can even be used to infer behaviour [31]. Dead-reckoning calculates the travel vector for a given time interval using information on heading, speed and change in the vertical axis [32]. Once this is achieved, the three dimensional movement path can be reconstructed by integrating the vectors in sequence [33–35]. Because data are recorded by sensors on board an archival logger, its efficacy is unaffected by the permissiveness of the environment [17, 18] which is important for obtaining accurate, unbiased data [36, 37]. In addition, archival loggers require considerably less power than GPS systems. Typically, a GPS running at 1 Hz may require between 30 and 50 mA of current, where as a modern iteration of the daily diary recording tri-axial acceleration and magnetometer data at 40 Hz requires only 5–10 mA of current (Holton, *pers. comm.*)

Dead-reckoning has been employed for tracking aquatic species [30, 33, 35, 38–40] but is yet to be used for species that utilise terrestrial locomotion. This is partly because of the difficulty for determining the speed of terrestrial animals [41], a process which is simpler underwater where mechanical methods can be used due to the density and viscosity of water [42–50]. However, an ability to estimate speed reliably for land animals should, in fact, make terrestrial dead-reckoning more straightforward than for aquatic or volant species [38] because terrestrial movement is not subject to drift due to air flow [51] or ocean currents [33]. Thus, the primary difficulty for terrestrial dead-reckoning may simply be the measurement of speed, and, were this to be provided, that this approach should provide a means to determine latent positions of animals between less frequent location data obtain by other means of telemetry [38].

Recently though, Bidder et al. [52] have shown that dynamic acceleration, as measured by animal borne inertial sensors, provides a means to estimate speed by proxy. Although the relationship between speed and dynamic acceleration can be perturbed by variations in substrate and incline [53], potential cumulative errors such as these [31, 38] could be corrected by periodic ground-truthing by a secondary means of telemetry. Indeed, this remains the most workable theoretical solution for terrestrial dead-reckoning, with the additional benefit that the use of accelerometers also enables behavioural analysis [54–56]. However, the terrestrial dead-reckoning method and the procedure for correcting tracks to verified positions has yet to be illustrated explicitly.

The present study details how terrestrial dead reckoning can be achieved using a novel correction method that couples accelerometer and magnetometer data to periodic ground-truths, obtained by a secondary means such as GPS telemetry.

## The dead-reckoning procedure for terrestrial animals

**Fig. 1:**
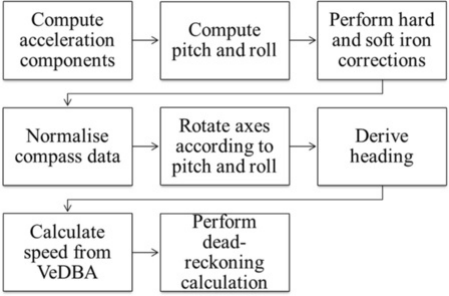
Flow diagram showing the process required to perform dead-reckoning

There are a number of stages required to obtain animal travel paths using dead-reckoning (Fig. 1), which require concurrent data from an animal-attached tag containing accelerometers and magnetometers with tri-axial orthogonal sensors recording at infra-second rates (e.g. typically >10 Hz). The stages are treated sequentially in detail below with brief discussion on potential system errors before the approach is trialled on animals to demonstrate performance.

### Computing acceleration components

**Fig. 2:**
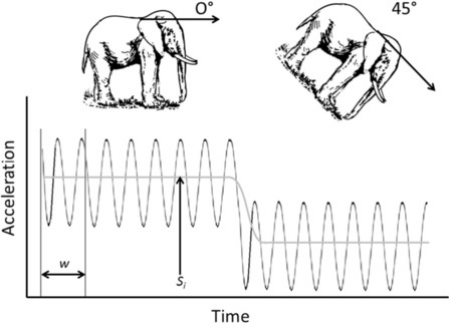
Idealised illustration of how static acceleration (S_i_) is calculated using a moving average of window size w. The locomotion of the animal produces a charicteristic waveform, which oscillates around the static acceleration S_i_

The ‘static’ acceleration [44, 57, 58] is required in order to undertake the necessary pitch and roll calculations for computing compass heading when the device orientation is not level, as is often the case when a tag is mounted on an animal. The static acceleration is that acceleration component due to the pull of gravity and amounts to 1 *g or* 9.81 ms^−2^ of acceleration and can be approximated using the method detailed in Shepard et al*.* [57], using a moving average (see Fig. 2).

The static acceleration for any sample, *S*_*i*_, given window size *w* may be computed using the equation given in Fang et al. [59];

$$ {S}_i = \frac{1}{w}\ {\displaystyle \sum_{j=i-\frac{w}{2}}^{i + \frac{w}{2}}}{S}_j $$

The dynamic acceleration (*DA*_*i*_*)* can be calculated by subtracting the static acceleration from the raw acceleration recorded by the accelerometer on each of the orthogonally placed axes (*x, y & z*). These values are then used to calculate VeDBA as described in Qasem et al. [60];

$$ VeDBA = \sqrt{\left(D{A}_x^2+D{A}_y^2+D{A}_z^2\right)} $$

This metric is used as a proxy for speed [53], below, while the static acceleration values are used to help calculate the attitude or pitch and roll of the device. Note that VeDBA values are the instantaneous measurements of dynamic acceleration for any given sample.

### Computing pitch and roll from accelerometers

Roll and pitch are calculated as rotations in the sway and heave (or surge) axes, respectively. For clarity, a tri-axial accelerometer records acceleration in the heave, surge and sway axes, corresponding to the dorso-ventral, anterior-posterior and lateral axes of the quadrupedal animal respectively [56]. If the static acceleration of heave, surge and sway are denoted by *S*_*x*_*, S*_*y*_ and *S*_*z*_ respectively, then pitch and roll can be calculated as (modified from [34]);

$$ Roll\ \left(\gamma \right)=\Big( atan2\left({S}_x,\ \sqrt{S_y \bullet {S}_y+{S}_z \bullet {S}_z}\right)\bullet \frac{180}{\pi } $$

$$ Pitch\ \left(\beta \right)=\Big( atan2\left({S}_y,\ \sqrt{S_x \bullet {S}_x+{S}_z \bullet {S}_z}\right)\bullet \frac{180}{\pi } $$

**Fig. 3:**
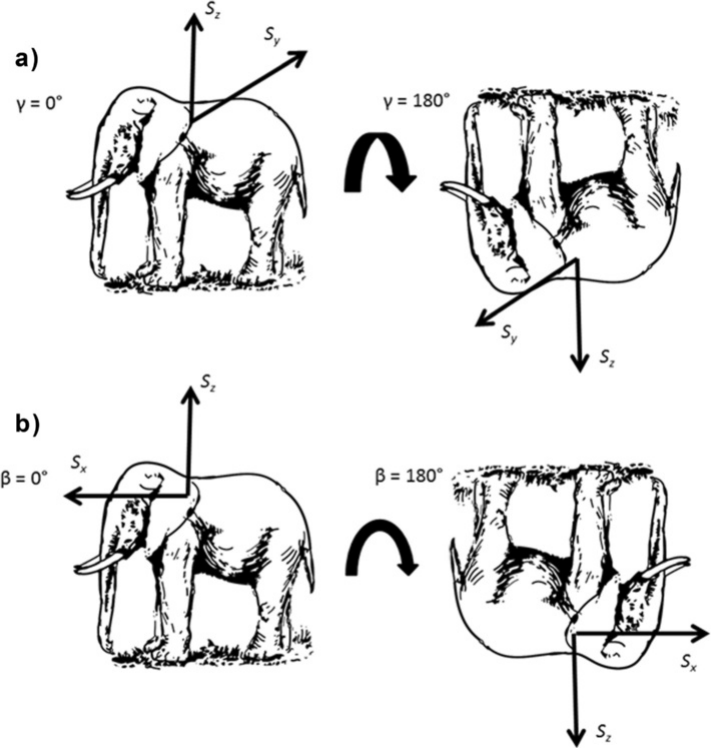
Illustration of how changes in body orientation, i.e. **a** Roll (γ), **b** Pitch (β), produce changes in static acceleration. S_x_ represents the Heave axis (dorso-ventral), S_y_ represents the Surge axis (*anterior-posterior*), and S_z_ represents the Sway axis (*lateral*)

This calculation normally provides pitch and roll in radians, so the presence of 180/π provides the result in degrees. The relationship between static acceleration and pitch and roll, is shown in Fig. 3.

Note that *atan2* is a function in computer programming, and is available in Excel, Matlab etc. and calculates the angle between the two coordinates given as arguments (separated by ‘,’) . In standard mathematical formula *atan2* may be expressed as;

$$ atan2\left(y,x\right)=2 \arctan \frac{y}{\sqrt{x^2+{y}^2}+x} $$

This proposed method calculates pitch and roll based on derivation of static acceleration using low-pass filtering based on a running mean, and is therefore prone to inaccuracies when animal movement is highly variable or sudden [61–64]. However, attitude is often measured using combined accelerometers and gyroscopes [59, 65–67] and certainly gyroscopes calculate attitude more accurately than accelerometers alone [62–64]. The question is whether this makes a real difference in dead-reckoning studies. Certainly, the limited difference in derived attitudes from accelerometers *versus* gyroscopes in wild animal studies [62–64] would imply not, especially since accelerometers alone reliably estimate attitude during periods of steady locomotory activity [59]. In addition, gyroscopes must record at very high sampling rates (>100 Hz), and have substantive power and memory requirements [63, 64] which precludes their use on many free-living animals with realistic package sizes and deployment periods [63, 64]. Indeed, it has been claimed that such inertial reference systems have weight, power requirement and costs that are tenfold those of simple accelerometer systems [68]. Other methods of deriving static acceleration from accelerometers without the need for gyroscopes exist, such as using a combination of Fast-Fourier transformation and low-pass finite impulse response filters [59, 69], or various other low-pass filters and approaches [44, 58, 70, 71]. We used the running mean method because it was already required to calculate the metric for dynamic acceleration, VeDBA [60] and because it has been demonstrably successful. Importantly though, the calculated static acceleration is only obtained to inform the orientation calculations, and the correction method for dead-reckoned tracks (see below) should filter any minor differences generated by using different static acceleration values in this stage of analysis.

### Hard and Soft iron corrections for magnetometers

The earth’s magnetic field can be distorted by the presence of ferrous materials or sources of magnetism near the magnetometer and this can result in errors in heading derivation due to the magnetometer’s susceptibility to magnetic distortions [72]. Few papers in the biological literature for dead-reckoning give explicit consideration to magnetic deviation in this manner [29–31, 33, 38], despite there being considerable discussion of its impacts within the engineering literature [73–76]. There are two primary sources of error in heading calculation from digital magnetometers; soft iron and hard iron magnetic distortions [77]. In the absence of magnetic distortions and after normalising the compass data for each axis, rotating the magnetometer through all possible orientations should produce a sphere when the data are plotted in a 3-dimensional scatterplot. This is because the magnetic field detected on each axis is the trigonometric product of the vector angle (i.e. heading) between them [78].

**Fig. 4:**
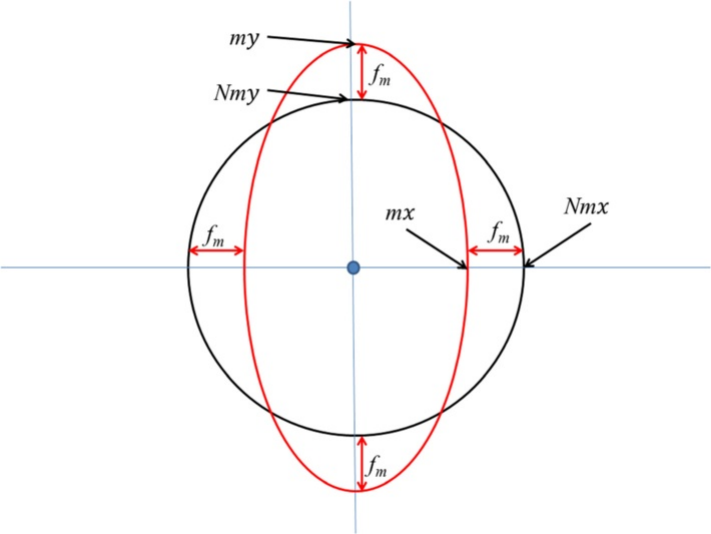
Visualisation of magnetometer data where distortions in the magnetic field due to soft iron sources near to the sensors may change the expected outputs on the various axes (two of three shown for simplicity). This can be corrected by appropriate normalising procedure (see text)

Soft iron distortions occur when ferrous material around the sensors (e.g. casing, screws, panels etc.), although not magnetic themselves (i.e. magnetically ‘soft’), alter the magnetic field around the device [68] causing the magnetic field to flow preferentially through them [79]. When a device containing tri-axial magnetometers is rotated under the influence of consistent soft iron distortions, the resultant data plotted in an X, Y, Z tri-axial magnetic field intensity plot (Fig. 4) are no longer a sphere but an ellipsoid, as the magnetic field observed by the sensors is dependent on the device’s orientation. These can be corrected by using an ellipsoid correction factor on the data before heading calculation provided that the position of the source of the soft iron distortion remains static relative to the movement of the magnetometer [80].

**Fig. 5:**
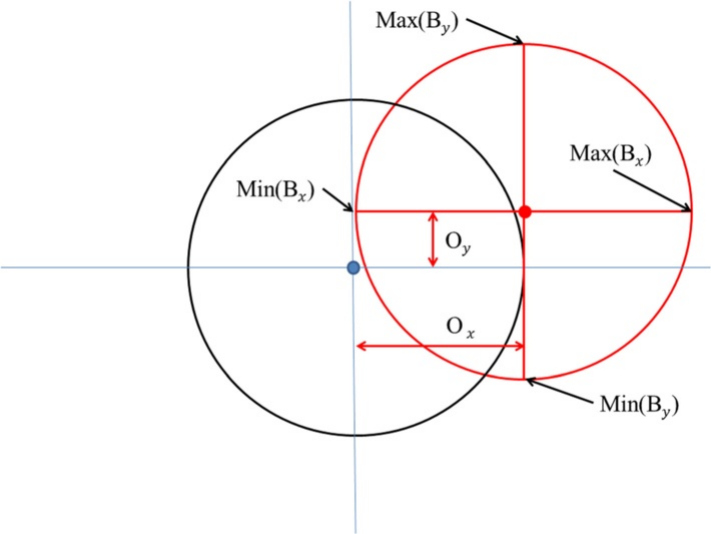
Visualisation of displacement correction for the magnetometer output in two axes. The red circle represents data for a 360° rotation from two magnetometer axes that are subject to displacement from the true point of origin (*black circle*) by hard iron distortion

‘Hard’ iron effects are caused by ferrous materials that have permanent magnetism, and thus their own magnetic field [81] and so add a constant magnetic field component that shifts the position of the centre of the sensor output. Correction applies a factor that returns the calibration to an origin of 0,0,0 [82] (Fig. 5).

To calibrate the device so that correction can be undertaken against both hard and soft iron distortions, the tag should be rotated through 360° whilst held level and then rolled 90° and rotated again. This process essentially allows each axis of the magnetometer to obtain values for the headings of North, East, South and West, denoted as *Bx, By* and *Bz*. As described in Merkel & Säll [83], for each axis, a minimum and maximum value are obtained, which is then used to calculate the offset produced by hard and soft iron distortions, denoted by *O* according to;

$$ {O}_x=\frac{ \max \left({B}_x\right)+ \min \left({B}_x\right)}{2} $$

$$ {O}_y=\frac{ \max \left({B}_y\right)+ \min \left({B}_y\right)}{2} $$

$$ {O}_z=\frac{ \max \left({B}_z\right)+ \min \left({B}_z\right)}{2} $$

This offset is then used to correct the output of the magnetometer to give the true magnetism experienced by the sensor on each axis, given as *m* via;

$$ {m}_x^h={B}_x-{O}_x $$

$$ {m}_y^h={B}_y-{O}_y $$

$$ {m}_z^h={B}_z-{O}_z $$

### Normalizing compass data

The compass data can be normalised using a normalising factor *f*_*m*_ [83];

$$ {f}_m = \sqrt{m{x}^2+m{y}^2+m{z}^2} $$

This factor is then applied to the outputs of each axis to normalise the magnetometer vector to unit length (Fig. 4) according to;

$$ N{m}_x=\frac{m_x}{f_m} $$

$$ N{m}_y=\frac{m_y}{f_m} $$

$$ N{m}_z=\frac{m_z}{f_m} $$

### Rotating axes according to pitch and roll

During device deployment the tag is unlikely to be kept level. This is problematic for the derivation of heading because tilting the device is alters the output of the magnetometer due to magnetic declination and inclination angles. Thus, pitch and roll values must be used to calculate what the magnetometer outputs were for the device to be orientated level, denoted by *RNm*_*x*_, *RNm*_*y*_ and *RNm*_*z*_;

$$ RN{m}_i=N{m}_i\bullet {R}_x\left(\gamma \right)\bullet {R}_y\left(\beta \right) $$

The rotation matrices (modified from [34]) for pitch and roll, given as *R*_*y*_(*β*) and *R*_*x*_(*γ*) respectively are expressed by;

$$ {R}_x\left(\gamma \right) = \left[\begin{array}{ccc}\hfill 1\hfill & \hfill 0\hfill & \hfill 0\hfill \\ {}\hfill 0\hfill & \hfill \cos \gamma \hfill & \hfill - \sin \gamma \hfill \\ {}\hfill 0\hfill & \hfill \sin \gamma \hfill & \hfill \cos \gamma \hfill \end{array}\right] $$

$$ {R}_y\left(\beta \right)=\left[\begin{array}{ccc}\hfill \cos \beta \hfill & \hfill 0\hfill & \hfill \sin \beta \hfill \\ {}\hfill 0\hfill & \hfill 1\hfill & \hfill 0\hfill \\ {}\hfill - \sin \beta \hfill & \hfill 0\hfill & \hfill \cos \beta \hfill \end{array}\right] $$

### Derivation of heading

The heading (*H*) in degrees may be calculated simply (see [84]) via;

$$ H=\left( atan2\left(RN{m}_y, - RN{m}_x\right)\right) \bullet \frac{180}{\pi } $$

### Calculation of speed from VeDBA

VeDBA (see above for calculation) is a good (linear) proxy for speed [52, 53] and can be incorporated together with speed (*s*) via;

$$ s = \left( VeDBA\bullet m\right)+c $$

where *m* is the constant of proportionality and *c* is a constant. During the dead-reckoning process (see below), the value for *m* can be changed iteratively until dead-reckoned paths and ground truth positions accord. In turn, speed (*s*) can be used to calculate distance, *d,* according to the time period length, *t*, as;

$$ d=s\bullet t $$

### Dead-reckoning calculation

To overcome Cartesian grid errors on a 3D Earth, it is necessary to determine a speed coefficient, *q,* using;

$$ q=\frac{d}{R} $$

where *d* is the distance for that time period and *R* is the radius of the earth (6.371 × 10^6^ m). Latitude and Longitude at time *T*_*i*_ can then be calculated via (sourced from Chris Veness, at http://www.movable-type.co.uk/scripts/latlong.html);

$$ La{t}_i=\mathrm{asin}\left( \sin La{t}_0\bullet \cos q+ \cos La{t}_0 \bullet \sin q \bullet \cos H\right) $$

$$ Lo{n}_i=Lo{n}_0+ atan2\left(\left( \sin H \bullet \sin q \bullet \cos La{t}_0\right),\ \left( \cos q - \sin La{t}_0\bullet \sin La{t}_i\right)\right) $$

### Verification of calculated paths

**Fig. 6:**
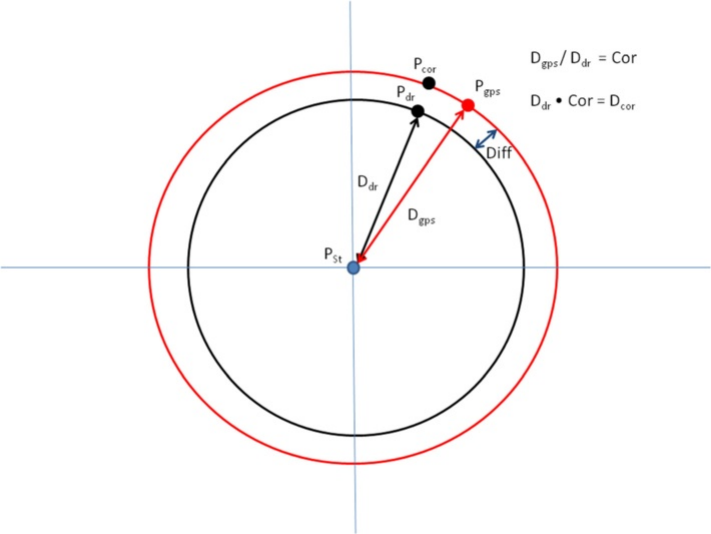
Illustration of how the distance correction factor is calculated. The correction factor is then applied to all distance calculations so that dead-reckoned and ground-truthed positions accord

In order to evaluate the accuracy of the calculated paths, synchronous ground-truthing data must be obtained. This can be achieved through the concomitant use of a secondary means of telemetry such as VHF or GPS. We used GPS, which produces a sequence of Latitude and Longitude values. When GPS and DD are perfectly synchronized, and assuming that the GPS estimates are perfect (but see later), the error in dead-reckoned (_*DR*_) position can be calculated by measuring the distance from a synchronous position obtained from the GPS via (sourced from Chris Veness, at http://www.movable-type.co.uk/scripts/latlong.html);

$$ Err=\mathrm{acos}\left( \sin La{t}_{DR}\bullet \sin La{t}_{GPS}+ \cos La{t}_{DR} \bullet \cos La{t}_{GPS} \bullet \cos \left(Lo{n}_{GPS}-Lo{n}_{DR}\right)\right)\bullet 6371 $$

where the distance between the two coordinates is given in km. If the coordinates do not accord, the dead-reckoned track can be corrected according to the following procedure; For any time period, the distance between consecutive GPS positions is first calculated. This distance is then divided by the corresponding distance for the same time period calculated by dead-reckoning, providing a correction factor by which dead-reckoning over- or underestimates speed. Subsequently, all speed values (*s*) for this time period can be multiplied by the correction factor (Fig. 6).

**Fig. 7:**
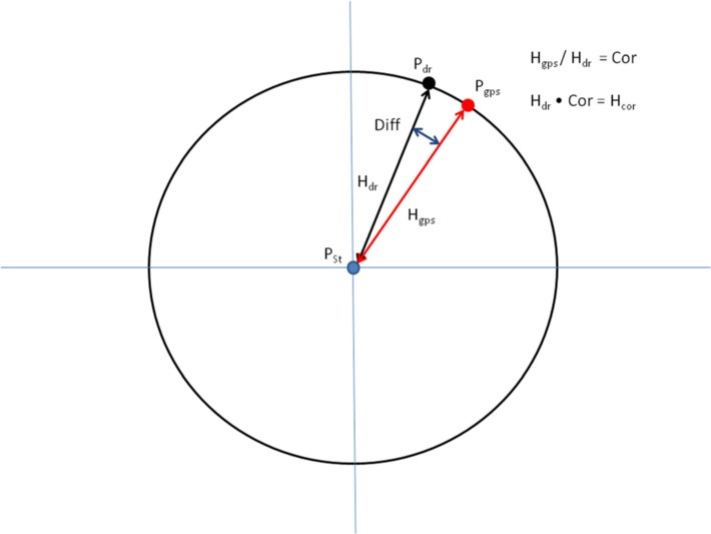
When distances between two time-synchronized positions derived from both GPS and dead-reckoned are equal but there is displacement between dead-reckoned and GPS-derived positions, a heading error is likely to have occurred but can be corrected (see text)

Non-accordance of the two tracks after this is indicative of a heading error, most likely due to the long axis of the tag imperfectly representing the longitudinal axis of the animal. To correct for this, the heading between the two ground-truthed positions that start and finish the relevant time period is calculated, as is the heading between the start and end positions of the dead-reckoned track. Then, in a manner similar to the correction of speed, the heading for the ground-truthed positions is divided by the heading for the dead-reckoned track to provide the heading correction factor (Fig. 7). This factor is applied to the heading data used in all intermediate dead-reckoning calculations and the dead-reckoned track then recalculated. This procedure of correcting distance and heading continues iteratively until dead-reckoned tracks and ground-truthed positions align.

## Results and Discussion

### Validation of terrestrial dead-reckoning

**Fig. 8:**
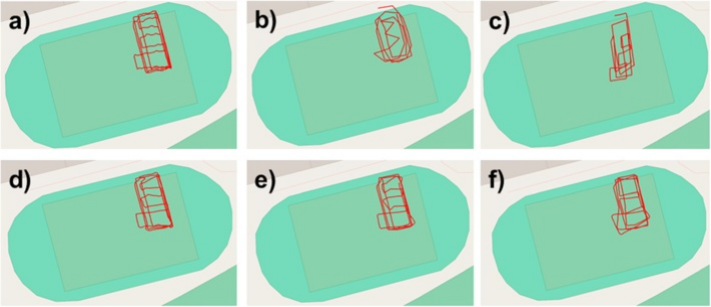
2D paths of the human participant as determined by **a** Video Recording, **b** GPS, **c** Dead-Reckoning without correction, **d** Dead-Reckoning with correction every 2 s, **e** Dead-Reckoning with correction every 5 s, **f** Dead-Reckoning with correction every 10 s

**Fig. 9:**
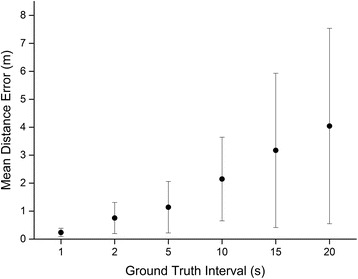
Mean Distance Error (m) at each of the ground-truthing regimes. Error bars represent Standard Deviation of the Mean

In order to validate the dead-reckoning technique we performed a simple experiment at Swansea University, UK. A human participant was equipped with a Samsung Galaxy S5 set to record GPS position every second and Accelerometer and Magnetometer data at 30 Hz. In addition, the participant’s position was logged simultaneously using a video camera. Geo-referenced points were obtained by performing a perspective transformation to acquire a top-down view. We manually labelled the position of the participant on each frame of the video, and then transformed the points using known reference positions to latitudinal and longitudinal coordinates. The participant walked a grid pattern within a 40 m by 20 m area on a grass playing field, in front of the camera’s field of view. The GPS data obtained was not sufficiently accurate in this instance to be used for ground-truthing the dead-reckoned track (see Fig. 8 panel b). The video positions were sub-sampled down to a position every 1, 2, 5, 10, 15 and 20 s and the dead-reckoning procedure was performed using each of these data sets for ground-truthing. The distance between the dead-reckoned positions during the latent periods (i.e. the periods between ground-truth correction) and the concordant video-derived positions (the complete, continuous data without sub sampling) was measured and termed the Distance Error. Figure 8 shows a comparison of the tracks dependent on the frequency of ground-truthing. GPS alone did not accurately reproduce the true pattern of movement at this scale. Figure 9 shows the mean Distance Error over the entire experiment at each of the ground-truthing frequencies. As expected, mean Distance Error increased with longer ground-truth intervals. Using a linear regression, we estimate the error accumulation rate to be 0.194 m per second between ground-truth corrections. At this stage, this figure is merely advisory as the error accumulation of terrestrial dead-reckoning is likely to be highly variable according to animal behaviour, surface type and track tortuosity [38, 53]. Further controlled trials are required to explore this issue. A video of this validation trial, comparing the track calculation according to different ground-truthing frequencies, is available in the supplementary information (see Additional File 1).

## Example studies on animals

The viability of the dead-reckoning procedure will be dependent on a large number of particularities (such as the terrain and the quality and frequency of the GPS fixes etc.) associated with the study animal in question. Thus, we present example results of dead-reckoning systems, deployed largely on domestic animals so as to be able to derive errors more readily, to give a general idea of the suitability of this procedure to determine terrestrial animal movements.

‘Daily Diary’ accelerometer loggers (wildbyte-technologies, Swansea, UK) and ‘i-gotU’ GPS data loggers (Mobile Action, Taipei City, Taiwan) were used to record both location and movement in a domestic dog (*Canis lupus familiaris*), horse (*Equus ferus caballus*), cow (*Bos Taurus*) and a badger (*Meles meles*) in deployment periods that lasted up to approximately 24 h. Loggers were attached using a leather neck collar to the badger and dog, to the saddle pad of the horse and by a surcingle-belt to the cow. Daily diaries recorded at 40Hz. GPS loggers recorded every 5 s for horse and dogs, 20s for cattle and every 60 min for badgers. Daily diaries weighed 28.0 g and had dimensions 46 × 19 × 39 mm. GPS loggers weighed 21.2 g and had dimensions 13 × 43 × 27 mm. The battery longevity for both the accelerometers and the GPS loggers was approximately 10 days.

### Dead-reckoning *versus* GPS

**Fig. 10:**
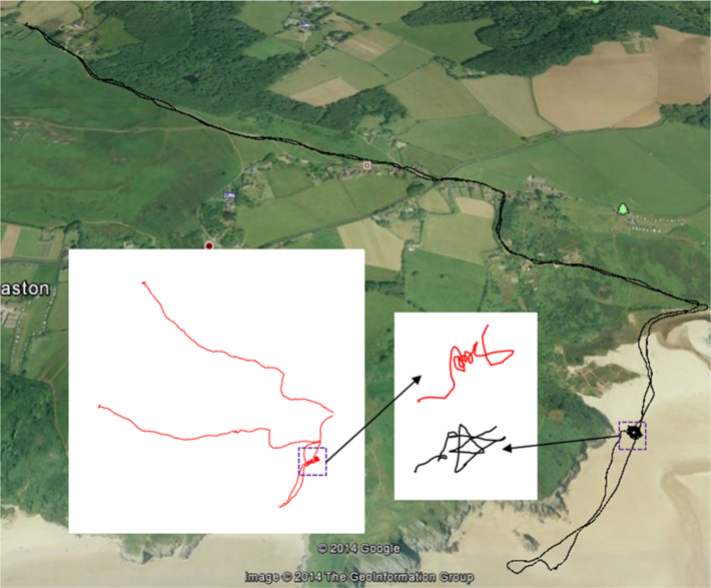
The movements of a rider-directed horse Equus ferus caballus, starting and ending in the *top left corner*, as elucidated by GPS (at 1 Hz - *black track*) and dead-reckoning (at 20 Hz) without any ground-truthed points (*red track*). Note that the dead-reckoned trace has no scale since the distance moved is derived from the speed and this is assumed to be linearly related to VeDBA, with a nominal relationship until ground-truthed (see text). The two dashed squares show a period when the horse was directed to move in tight circles. For scale, the total track length according to the GPS (*black track*) was 10.127 km

Dead-reckoning- and GPS-derived positions are fundamentally different but give superficially similar results (Fig. 10). GPS-derived positional data show excellent spatial coherence at scales over 10 m and are independent of time. Dead-reckoned tracks replicate the major features shown by GPS-derived tracks but, when they have no ground-truthed points along them, are uncoupled from the environment and generally show decreasing coherence with respect to themselves over time (Fig. 10). Nevertheless, over small time and scale intervals, dead-reckoned data show features in movement that are often lost in GPS-derived positions. For example, during data acquisition used for Fig. 8, the horse was directed to move in tight circles, which are much better resolved than the GPS data.

### GPS-enabled dead-reckoning

The value of using GPS to correct for inaccuracies in dead-reckoned trajectories can be illustrated using GPS-enabled DDs [38] on domestic dogs *Canis lupus familiaris* and horses. Here, we provided conditions where the dogs could move ‘freely’, as they accompanied their owners, while the horse movement was directed by a rider. In these cases, there was variable discord between GPS positions and dead-reckoned positions for both the dogs and the horse (Table 1). However, calculation of animal travel paths, determining speed according to VeDBA without any form of correction, unsurprisingly, produced appreciable error (Mean = 732 ± 322 m). Once corrections for distance and heading were applied, average distance between GPS and dead-reckoned positions was negligible though (Mean 0.29 ± 0.2 m).

**Table 1:** Details for accordance between GPS and dead-reckoned positions prior to and post correction procedure for data derived from animals using DDs recording tri-axial acceleration and tri-axial magnetic field strength, with 12-bit resolution, at a sampling frequency of 20 Hz with GPS loggers (iGotU GT-120, Mobile Action Technology) recording at 0.2 Hz. Devices (61 g) were collar-mounted in the dogs, and placed on the saddle pad above the withers in the horse

Subject	Mean Error before correction (m)	Mean Error after correction (m)
Dog 1	591	0.13
Dog 2	653	0.11
Dog 3	480	0.53
Horse	1204	0.39
Mean	731.0	0.29
St. Dev	322.5	0.20

In addition, after correction, the total distance apparently travelled by the animals differed according to whether it was calculated by dead-reckoning or GPS, with dead-reckoned tracks being consistently higher (Table 2) and mean differences between measures of total distance for GPS and dead-reckoning being 0.702 ± 0.465 km. This highlights the effect of enhanced tortuosity displayed by dead-reckoned paths, even when the GPS-derived positions are being recorded at 0.2 Hz.

**Table 2:** Summary of track length estimations according to GPS telemetry and dead-reckoning for data presented in Table 1. The tortuosity was calculated as average change in heading between measurements (at 40 Hz)

Subject	GPS Length (km)	Dead Reckoning Length (km)	Difference (km)	Tortuosity (°)
Dog 1	5.507	6.410	0.902	6.38
Dog 2	2.075	2.492	0.418	6.16
Dog 3	3.040	4.296	1.256	11.52
Horse	10.127	10.360	0.233	4.38

**Fig. 11:**
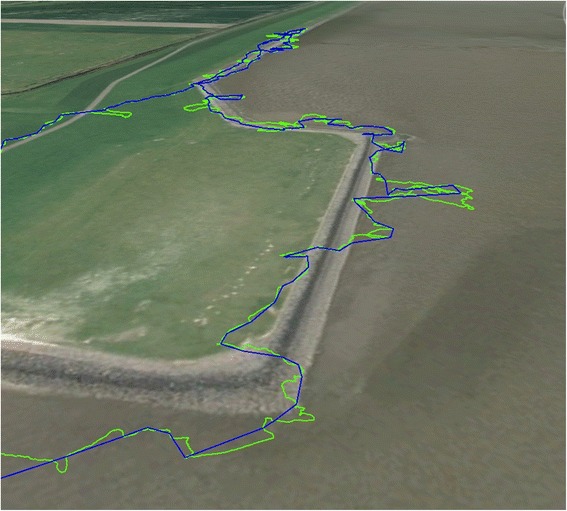
Movement path of a domestic dog. The purple track displays the GPS data (at 0.2 Hz) while the green shows the dead-reckoned path (at 40 Hz). For scale, the total track length according to the GPS was 3.040 km. Note the additional track tortuosity of the dead-reckoned track

**Fig. 12:**
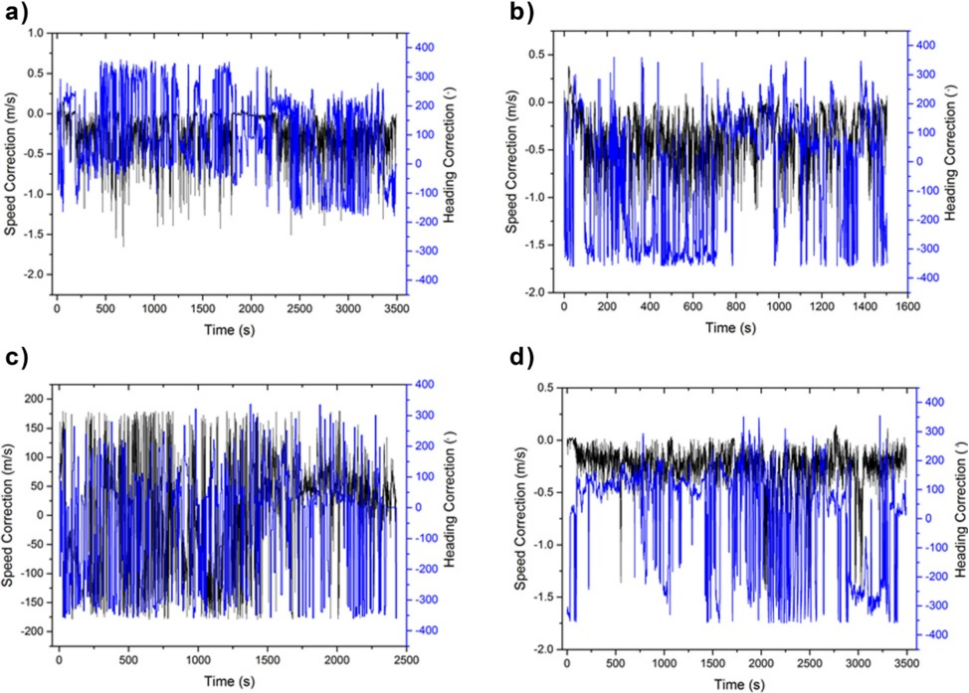
Changes in speed and heading correction factors necessary to tie dead-reckoned tracks into those acquired by GPS during deployment of a GPS-enabled DD on a) dog 1, b) dog 2, c) dog 3 and d) a horse. Note that the speed correction value changes relatively little but the heading estimates sometimes varied considerably

The difference in poorly, *versus* highly, defined tracks derived from just GPS and GPS-enabled dead-reckoned trajectories was most apparent in the dogs (Table 2), presumably because, due to their smaller size and active behaviour, dogs were much more likely to deviate from the course defined by the GPS trajectory within any 5 s period of time (Fig. 11). The dead-reckoned track highlights the high degree of tortuosity in the tracks (Fig. 11) but also explains why there was appreciable variation in the correction factors required to tie in the derived speed and heading for dead-reckoned tracks with the GPS positions over time (Fig. 12).

In particular, although there was relatively little variation in the speed correction factor over time, the heading correction was, at times, radically different from that initially computed using the acceleration- and magnetometry-derived data (Fig. 12). The cause for this is due, in part to the high frequency of GPS fixes, the error in those fixes and the speed of the animal. Another possible source of errors may be associated with the estimation of static acceleration (used in the orientation correction of magnetometer data) via smoothing of the data. Further studies should be able to assess this by using more than one method to estimate static acceleration and monitoring if heading estimations differ.

**Fig. 13:**
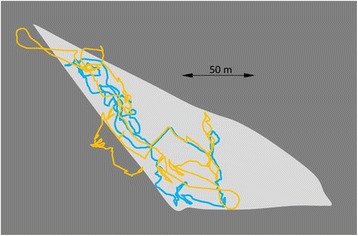
GPS-enabled dead-reckoned tracks (DD sampling rate 40 Hz, GPS sampling rate 0.1 Hz) from a domestic cow Bos taurus in an enclosed field (l*ight grey area*) over 2 h. The yellow track shows the calculated trajectory using all GPS points (at 20 s intervals) while the blue track shows the calculated trajectory omitting all unrealistic GPS points (based on speed and estimates outside the peripheral fence). Note that various elements of the GPS, such as the Kahlmann filter, may give highly credible loops within the track that do not correspond to the real trajectory of the animal (cf. *yellow lines* outside the field periphery)

The effect of the errors in the GPS positions on dead-reckoned tracks and the correction factors required to tie these in with GPS positions is likely to increase with increasing GPS sampling frequency because, even though GPS positional estimates are relatively small (of the order of a few metres [18–20, 85]), these become relatively larger as the scale over which movement is considered decreases (Fig. 13).

This highlights why temporally finely resolved GPS positional estimates require such radical heading corrections in headings derived using dead-reckoning. In slow-moving animals such as cows (Fig. 13), GPS fixes can be calculated as being several metres in front of the true position and several metres behind in an animal that is moving slowly in one direction. In such a case, corrected dead-reckoned tracks will, at times, have to use a heading that is the exact opposite of that derived using the magnetometry data. This problem will presumably diminish as GPS fixes become less frequent and as the speed of the animal increases.

**Fig. 14:**
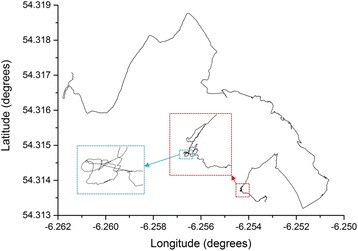
GPS-corrected (30 min intervals,) dead-reckoned (40 Hz) track of a wild badger Meles meles over 200 mins during which time the animal was calculated to have moved to a distance of 670 m from its sett. The boxes show zoomed sections of the track to illustrate the effective maintenance of resolution over even short time intervals

All this emphasises the value of dead-reckoning *per se*, in helping define very fine animal movements (Fig. 14), where such definition may even help identify animal behaviours although such data may not be in perfect spatial placement. Otherwise, dead-reckoning is clearly useful for filling in likely trajectories for animals where positional information via GPS is only gained infrequently although the errors will require much more work to formalise. However, this work points to appreciable problems that will need to be resolved when GPS-based positional information is acquired at high frequencies and dead-reckoning is to be used to derive a trajectory. One approach is to filter GPS point accuracy according to the number of satellites used to derive the positional fix, or similar metrics such as minimum distance or motion sensor threshold [86]. However, even this will never give perfect spatial resolution. A better way forward may be to combine such metrics with an error circle and consider the extent to which the dead-reckoned path may pass through it.

### Potential and pitfalls in ground-truthed terrestrial dead-reckoning

This exploratory work suggests that dead-reckoning is a viable means to track terrestrial animal movements on a fine scale. One notable advantage of terrestrial, compared to fluid-based dead-reckoning, is that there is no drift due to horizontal vectors such as wind or currents [38]. However, dead-reckoned data must be periodically ground-truthed because system errors, such as imperfect tag orientation on the animal and terrain effects [52, 53], cause the track to become uncoupled from the environment. The appropriate frequency and quality of such ground-truthed points is complex. GPS loggers used on wild animals typically record at fix at periods ranging over seconds [87–89], hours [90, 91], days [92] or even months [93] and have errors that depend on the permissiveness of the environment [18–20, 85] so a clear next stage in this work is to derive a rule book for maximizing the value of both GPS and dead-reckoned data according to the questions being asked. In environments in which accurate GPS locations are not possible (e.g. under dense canopy cover [17, 20, 94–98]) ground-truthing may be more reliably achieved using Radio Frequency Identification (RFID) stations or camera traps at known locations.

### Implications of dead-reckoned tracks for understanding movement ecology

While the advantages of GPS-derived data are clear, those of dead-reckoned data have received less attention, perhaps because of the limited number of users. Importantly though, dead-reckoned data show relative movement with very fine resolution, with better coherence of these data the closer they are in time to each other. With the advent of novel open-source analysis software (see [99], in this volume), dead-reckoning may also be implemented with little computational acumen or programming skill. Thus, we expect researchers to be able to use movement defined by dead-reckoned tracks over seconds to be able to resolve behaviours, examining 2- or 3-d space use as a template in the same manner as accelerometry data [56].

Currently, the majority of studies of animal movement are based on infrequent positional fixes obtained via transmission telemetry, calculating distance by assuming straight line travel between fixes [100]. The assumption of such straight line paths ignores sub-fix tortuosity in animal paths [101] and leads to obvious underestimations of animal travel distance, both theoretically (Fig. 2; [102–104]) and practically [100, 105–107]. Such issues become critical for examining models such as Lévy Walk, where animal movement should be scale-independent (see [108] and references therein) and needs to be examined as such [85, 109]. Solutions require sampling animal position with higher temporal resolution [110–112], which may be possible for larger animals [86, 109] with improvements in GPS technology [113] but, ultimately, fixes need to resolve the minimum radius tortuosity [100].

The GPS-enabled dead-reckoning method described in the current study is the only method by which distance and animal tortuosity can be measured accurately independent of any bias due to scale [31, 38]. Not only should this method provide new information on the habits of animals, but it offers a means for acquiring data on, and testing recent theoretical developments in movement ecology such as Correlated Random Walks, Levy Flights and State-space models (c.f. [114–122]). Given that tortuosity and movement patterns are likely to vary between species, populations and individuals, this new tool available to animal ecologists may be the only means to measure this variation properly, and should be considered a significant development in the understanding of movement ecology [100, 123].

## Conclusions

Dead-reckoning has the potential to record the fine scale movement of terrestrial animals. To obtain the same level of detail from GPS telemetry alone, devices would require large amounts of power and could induce bias at small scales. Despite dead-reckoning having been employed on aquatic species, numerous methodological barriers restricted its use on terrestrial species. This study is the first explicit demonstration of terrestrial dead-reckoning and should provide adequate information to be used by those researchers of terrestrial species that are currently limited to temporally sparse GPS telemetry. These continuous, fine scale dead-reckoned tracks should record animal movement on a step by step basis, providing a complete account of animal location and movement. Initially, estimation of speed for integration in dead-reckoning calculations was problematic for terrestrial animals, but this issue has been largely overcome by use of accelerometers and a novel correction method that makes use of secondary ground truth positions. This technique has the potential to develop our understanding of animal movement ecology, and inform movement models that better reflect the true nature of animal movement patterns.

## Availability of supporting data

A video detailing the dead reckoning validation experiment and illustration of results depending on different correction regimes is available in Additional File 1.

## Additional file

Additional file 1:
**Video detailing dead reckoning validation experiment and illustration ofresults depending on different correction regimes.** (MP4 14701 kb)
